# Coping Strategies and Psychological Maladjustment/Adjustment: A Meta-Analytic Approach with Children and Adolescents Exposed to Natural Disasters

**DOI:** 10.1007/s10566-022-09677-x

**Published:** 2022-02-20

**Authors:** Daniela Raccanello, Emmanuela Rocca, Veronica Barnaba, Giada Vicentini, Rob Hall, Margherita Brondino

**Affiliations:** 1 Department of Human Sciences, University of Verona, Lungadige Porta Vittoria 17, 37129 Verona, Italy; 2 Macquarie University and Environmetrics, Sydney, Australia

**Keywords:** Coping strategies, Maladjustment, Adjustment, Natural disasters, Children and adolescents, Meta-analysis

## Abstract

**Background:**

Following disasters, children and adolescents can use coping strategies to feel better. A growing body of studies investigated the relation between them and maladjustment/adjustment, i.e., negative symptomatology/positive indicators of development. Yet, these constructs are studied separately.

**Objective:**

We conducted two meta-analyses to examine the mean correlation between disaster-related coping strategies and indicators of maladjustment/adjustment following natural disasters in children and adolescents, considering the role of some moderators.

**Methods:**

We used PsycINFO, PubMed, Eric, and Scopus databases to identify articles on natural disasters (filters: participants ≤ 18 years at the disaster, peer-review, English language). Inclusion required investigating the relation between at least one coping strategy and at least one indicator of maladjustment (e.g., post-traumatic stress disorder, depression) and/or adjustment (e.g., self-efficacy, emotion understanding), for a total of 26 studies (*k* = 64, *n* = 9692, for maladjustment; *k* = 37, *n* = 3504, for adjustment).

**Results:**

There were global positive significant correlations between coping strategies and negative symptomatology (*r*_*pooled*_ = .23) for maladjustment, and positive indicators (*r*_*pooled*_ = .17) for adjustment. Negative symptomatology positively correlated with escape (*r* = .19), social isolation (*r* = .15), submission (*r* = .64), and opposition (*r* = .16); positive indicators positively correlated with problem solving (*r* = .31), social support (*r* = .22), and submission (*r* = .30). We found a moderating role of age, disaster type, and continent for maladjustment.

**Conclusions:**

The study presented an analysis of the coping strategies that can be effective for children and adolescents dealing with natural disasters.

## Introduction

Natural disasters such as earthquakes, floods, or fires seem to be occurring at an increasing rate, sometimes leading to catastrophic situations that suddenly disrupt everyday life, resulting in great damage and destruction (Assar, 1971; EM-DAT, n.d.; Fergusson & Boden, 2014). They can cause great suffering, destroying people’s physical, biological, and social environment, with short and long-term consequences for their health and wellbeing (World Health Organization, n.d.). Their impact is particularly relevant for children and adolescents, given their vulnerability related to their cognitive and emotional level of development (Kar, 2009; Masten & Osofsky, 2010). Little is known about the efficacy of coping strategies when dealing with the traumatic consequences of disasters; nevertheless, this knowledge is a necessary step in identifying strategies for preparing children and adolescents to face possible future disasters. Therefore, we examined the literature on natural disasters focusing on the relation between coping strategies and psychological maladjustment/adjustment in children and adolescents using a meta-analytic approach.

### Impact of Natural Disasters on Children and Adolescents

Many studies on the effects of being exposed to large-scale traumatic events document the impact of these events on psychological maladjustment; however, some studies also point to the existence of increased resilience through adjustment (for examples of measures on psychological maladjustment/adjustment see Cheng et al., 2014).

The exposure to natural disasters can lead to serious negative short and long-term effects for children and adolescents (Fergusson & Boden, 2014; Furr et al., 2010; Kar, 2009; Masten & Osofsky, 2010; Neria et al., 2008; Tang et al., 2014; Wang et al., 2013; Weissbecker et al., 2008). Compared to adults, children and adolescents’ vulnerability to the deleterious effects of disasters is due to the fact that they are less well equipped with adaptive coping strategies and abilities to control emotions such as anxiety (Norris et al., 2002; Weems et al., 2015). Maladjustment includes traumatic consequences for mental health, with increased rates of psychopathology such as post-traumatic stress disorder (PTSD), depression, anxiety and fear, and psycho-social distress, usually with a peak of symptoms or effects in the first year after the disaster.

A meta-analysis including more than 74,000 children and adolescents who were victims of natural or technological disasters indicated that the magnitude of the association between disasters and PTSD, ranging from small to medium, varied according to factors such as the characteristics of the children (e.g., gender) or the characteristics of the exposure (e.g., disaster type; Furr et al., 2010). However, data on the prevalence and the severity of symptoms are inconsistent, with decreasing, increasing, or stable levels over time (Kar, 2009). For example, Kar (2009) reported that the prevalence of PTSD varied between 5 and 43%, while a meta-analysis involving more than 42,000 participants indicated variations between 1 and 95% (Wang et al., 2013). Also figures on the prevalence of depression vary widely, ranging for example from 1.6 to 81% (Wang et al., 2013), from 7.5 to 45% (in a meta-analysis with more than 12,000 children, Tang et al., 2014), or from 2 to 69% (in another meta-analysis with four-to-17-year-olds, Lai et al., 2014). A variety of risk factors for depression have been investigated. These include age (with inconsistent findings about the expected higher prevalence amongst older compared to younger children), trauma characteristics such as being entrapped or witnessing injuries or deaths during the disaster, and post-trauma characteristics such as the absence of social support (Lai et al., 2014; Tang et al., 2014). Bonanno et al. (2010) reported that exposure to disasters can increase the probability of experiencing anxiety disorders, although published meta-analyses such as that by Wang et al. (2013) found relatively few studies that explored this relationship. Finally, psycho-social distress can follow a disaster, as found for example amongst children and adolescents who were victims of hurricanes or earthquakes (Vigil & Geary, 2008; Vigna et al., 2010; Yang et al., 2010).

Even if maladjustment is quite common in children and adolescents who experienced a disaster (Flynn & Norwood, 2004), some studies indicated that exposure could in some cases represent a positive “turning point experience” (Rutter, 1996) and that post-disaster sequelae could include psychological adjustment. Adjustment can be assessed through a variety of measures (Cheng et al., 2014) pertaining to cognitive, emotional, social, or motivational domains. Notwithstanding that psychological distress may impact children and adolescents’ cognitive and emotional functioning, some studies report that using particular coping strategies after disasters such as earthquakes is associated with increases in cognitive performances (Cadamuro et al., 2015). In addition, one study showed that two years after an earthquake there were no differences in emotion regulation and understanding between children who had been victims of the disaster and a control group (Raccanello et al., 2017). Concerning the social domain, a few studies reported positive effects of exposure to natural disasters, such as a better understanding of themselves and others (Yang et al., 2010), the growth of communication and relationship skills (Bokszczanin, 2012), as well as attention, care, and altruism (Benenson et al., 2007). Other studies reported that post-traumatic stress symptoms were positively associated with intention to contact and help other survivors (Vezzali et al., 2016). Li et al. (2013) found that age impacted the tendency to be altruistic after witnessing disruption due to a major earthquake: While nine-year-olds increased altruistic giving, six-year-olds became more selfish. However, these differences vanished after three years. Regarding the motivational domain, some studies on the effects of hurricanes and earthquakes reported that perceived competence, self-efficacy, or self-concept can play a key role for post-disaster adjustment (Cryder et al., 2006; Kilmer & Gil-Rivas, 2010; Wu et al., 2014; Yang et al., 2010). Finally, some authors reported that child victims of tornados were more or less resilient in the face of adversity, with variations in recovery depending on the presence of protective factors such as self-regulation or returning to school (Vezzali et al., 2016). Overall, increases in resilience and adjustment reflect effective coping and adaptation in the face of major life stress (Masten & Osofsky, 2010).

### Risk and Protective Factors

Research findings indicate that children and adolescents’ reactions and susceptibility to natural disasters can vary in response to a large number of factors, related to biological, psychological, and contextual dimensions (Masten & Osofsky, 2010; Weems, 2015). This variability is reflected in the various estimates reported in the data on the prevalence of post-traumatic reactions and by the fact that notwithstanding having experienced a trauma, a percentage of children and adolescents develop resilience (Kilmer & Gil‐Rivas, 2010; La Greca et al., 2002).

First, findings concerning age differences are not consistent for reactions such as antisocial and aggressive behaviors or PTSD (Celebi Oncu & Metindogan Wise, 2010; Vezzali et al., 2016). The different levels of vulnerability between children and adolescents could be related to different risk and protective factors (Masten et al., 1990). Younger children are frequently protected by a lower exposure to disaster, especially when caregivers and people supporting them remain stable (Osofsky, 2004; Silverman & La Greca, 2002). Adolescents are more exposed both to disaster-related information and to risks given their higher involvement within the society, but at the same time they have more resources, such as problem solving skills, social support outside the family, or survival skills to cope with negative events (Masten & Osofsky, 2010).

Second, natural disasters differ in characteristics such as causes, frequency, controllability, rapidity of onset, duration of the alarm and emergency phases, extension of the area of impact, disruptive potential, duration of the following risk, and probability of reoccurrence of the event (Cuzzolaro & Frighi, 1991). Therefore, it is plausible that these aspects can impact psychological functioning in different ways. Specifically, some studies indicated that the consequences of human-induced compared to natural disasters are more likely to persist over time (Green et al., 1992). Another study found that the type of disaster (e.g., natural, technological) is a weaker predictor of children’s PTSD compared to the extent of their exposure to the disaster (Celebi Oncu & Metindogan Wise, 2010).

It is worth noting that some studies examined the role of the time elapsed since a disaster (e.g., Wang et al., 2013). While traumatic and negative reactions are typically more intense in the first year after a disaster, they do not always decrease over the long-term (Celebi Oncu & Metindogan Wise, 2010; Gökçen et al., 2013; Kar, 2009; La Greca et al., 1996; Raccanello et al., 2017). Data on adults indicate that adjustment measured, for example, in terms of optimism did not vary after several months (Prati & Pietrantoni, 2009).

### Coping Strategies

Following disasters, children and adolescents can use a large variety of coping strategies to feel better. Coping is a multi-component construct referring to all the ways employed to face stressful events (Skinner et al., 2003). In the literature, different classifications of coping strategies have been proposed. Among them, the pioneering work of Lazarus and Folkman (1984) distinguished between problem and emotion-focused strategies; the first oriented to find a solution to the problematic event that caused the negative emotions, and the second to alleviate the distress caused by it. Schaefer and Moos (1992) differentiated active and avoidant coping, focused respectively on actions to approach the problem with processes of cognitive reconstruction, elaboration, and support seeking on the one hand, and ways to escape from the problem, feeling helpless, and trying to obtain alternative rewards on the other hand. Attempting to produce a broad classification of coping strategies, taking into account age changes, Zimmer-Gembeck and Skinner (2011) proposed a developmental classification in which they code strategies into three categories corresponding to different adaptive functions, focused on competence, relatedness, and autonomy (Deci & Ryan, 1985). Each category included two connected families of strategies and their opposites. The first set, focused on competence, involves problem solving and information seeking in contrast to helplessness and escape. These strategies help individuals to adapt their behaviors to the environmental constraints they face. The second set, focused on relatedness, comprises self-reliance and social support in contrast to delegation and social isolation. It revolves around endeavors to build reliance amongst and between people caught up in the situation. The third set, focused on autonomy, includes accommodation and negotiation in contrast to submission and opposition. These strategies are organized around efforts to “trade” options to reach one’s own goals.

Some meta-analyses have investigated the effects of using different coping strategies on stressful events. For example, passive coping strategies such as avoidance, social isolation, and rumination, have been found to have negative effects on the psychological adjustment to stressful events (Cofini et al., 2015). A meta-analysis focused on the violence that women suffer from their partners revealed that escape as a type of strategy, seems to facilitate the maintenance of the disorder, and is positively related to PTSD symptoms. On the other hand, Clarke (2006) explored the relation between active coping and psychosocial health among youth and confirmed that active coping strategies such as problem solving, assertive communication, and seeking social support are linked to healthy adjustment (Compas et al., 2001; Fields & Prinz, 1997; Grych & Fincham, 1997). However, it is worth noting that the adaptivity of different coping strategies depends on the characteristics of the individuals and of the context.

Concerning disasters, a growing body of studies is exploring the relation between coping strategies and children’s maladjustment/adjustment. These various studies investigated coping types such as problem-focused vs. emotion-focused coping, active vs. avoidance coping. Some studies focused on the relation between coping strategies and negative symptomatology such as PTSD, depression, and anxiety after exposure to a natural disaster (e.g., Papadatou et al., 2012; Terranova et al., 2009; Vigil & Geary, 2008). For example, Tang et al. (2014) conducted a meta-analysis on risk factors for depression in children and adults who survived natural disasters, observing that the most significant predictors for children were having experienced previous trauma, being trapped or injured during the disaster, having witnessed injury or death during the disaster, and not receiving social support. Findings from Weems and Graham (2014) indicated that, in the context of hurricanes, more resilient children—characterized by low post-traumatic stress symptoms and high exposure—have lower levels of avoidance coping compared to the others. Some studies revealed an association between coping strategies and adjustment in terms of resilience, self-concept, and self-efficacy (e.g., Bokszczanin, 2012; Stratta et al., 2013; Wang & Gan, 2011). A meta-analysis by Prati and Pietrantoni (2009) investigated the relation between some coping strategies and indicators of adjustment such as the post-traumatic growth (PTG) in adults. The results showed that strategies such as optimism and social support are positively related to PTG.

In summary, the psychological literature suggests that different coping strategies can vary in their efficacy from case to case when applied in natural disasters. However, the role of coping strategies as risk or protective factors in relation to disasters has not yet been examined systematically. It is worth noting that most of the interventions examined in the psychological literature were conducted after a disaster has occurred (for meta-analyses on their efficacy see Brown et al., 2017; Kar et al., 2009; Pfefferbaum et al., 2019). However, disaster preparedness and prevention are of key relevance to support adjustment and use of effective coping strategies in the case of an emergency (for example of one intervention conducted before disasters, see Raccanello, et al., 2019b, 2020a, 2021; Vicentini et al., 2020).

### Current Study and Hypotheses

Using a meta-analytic approach, we synthesized the body of research on disaster-related coping strategies with children and adolescents. We aimed at assessing the mean correlation between several coping strategies (Table 1; Zimmer-Gembeck & Skinner, 2011) and maladjustment/adjustment measures, following natural disasters, taking into account the role of some moderating factors.

**Table 1:** Overview of the selected studies

Author/s (year)	Journal	Type of disaster	Date of disaster	Months after disaster	Country (continent)	N (% F)	Age (range or mean age in years)	Type of coping strategy	Instruments for coping strategies	Maladjustment/adjustment measures	Category of maladjustment/adjustment	Instruments for maladjustment/adjustment	Study number for forest plots (Fig. 3, M = maladjustment; Fig. 5, A = adjustment)
An et al. (2013)	Journal of Loss and Trauma: International Perspectives on Stress & Coping	Earthquake	May 12, 2008	24	China (Asia)	636 (62%)	13–16	Escape	Coping Style Scale (Xiao & Xu, 1996)	Maladjustment	PTSD	Child PTSD Symptom Scale (Foa et al., 2001)	M1
Andrades et al. (2018)	Journal of Happiness Studies	Earthquake	February 27, 2010	12	Chile (America)	172 (53%)	10–15	Submission	Rumination Scale for Children (Cryder et al., 2006)	Maladjustment, Adjustment	PTSD, General	The Posttraumatic Growth Inventory for Children-Revised (PTGI-C-R; Kilmer et al., 2009)	M61
Bokszczanin (2012)	Anxiety, Stress & Coping	Flood	July, 1997	21	Poland (Europe)	262 (37%)	13–21	Social support	Proactive Coping Inventory (PCI; Greenglass et al., 1999)	Adjustment	Social	Inventory of Postdisaster Social Support (Norris et al., 2001); Perceived social support (Czapinski, 1998)	A30, A31, A32
Cadamuro et al. (2015)	Child & Youth Care Forum	Earthquake	May, 2012	6	Italy (Europe)	517 (51%)	7–12	Escape	Kidcope (Spirito et al., 1988)	Adjustment	Cognitive	Second-Order False Belief Tasks: Birthday Puppy (Sullivan et al., 1994), Double Bluff (Happé, 1994); Comprehensive Test of Nonverbal Intelligence (Hammill et al., 1996); Raven’s Standard Progressive Matrices (Raven, 1981)	A1, A2, A24
Cryder et al. (2006)	American Journal of Orthopsychiatry	Hurricane	September 7, 1999	12	USA (America)	46 (61%)	6–15	Problem solving	The Children’s Competency Beliefs Scale, adaptation of the Child’s Coping Efficacy Scale (Weyer & Sandler, 1998)	Adjustment	General	The Posttraumatic Growth Inventory for Children (PTGI-C), adaptation of the Posttraumatic Growth Inventory (Tedeschi & Calhoun, 1996)	A3, A18
Felton et al. (2013)	Journal of Abnormal Psychology	Flood	May 3, 2010	6	USA (America)	227 (56%)	10–15	Accommodation, Submission	Response Style Questionnaire (RSQ; Nolen-Hoeksema & Morrow, 1991)	Maladjustment	Depression	Children’s Depression Inventory (CDI; Kovacs, 1992)	M2, M3
Kilmer and Gil‐Rivas (2010)	Child Development	Hurricane	August 23, 2005	12	USA (America)	66 (56%)	7–10	Problem solving, Social support, Submission	Children’s Competency Beliefs Scale (CCBS; Weyer & Sandler, 1998); Caregiver warmth and acceptance (Greenberger & Chen, 1996); Rumination Scale for Children (RS-C), adaptation of the Adult Rumination Scale (Calhoun et al., 2000)	Maladjustment, Adjustment	PTSD, Motivational, General	Child Post-Traumatic Stress Symptoms (UCLA-PTSD RI-1; Steinberg et al., 2004); Self-Perception Profile for Children (Harter, 1982); The Posttraumatic Growth Inventory for Children-Revised (PTGI-C-R; Kilmer et al., 2009)	M4, M5, M6 A4, A7, A8, A9, A13, A19, A20, A21
La Greca et al. (1996)	Journal of Consulting and Clinical Psychology	Hurricane	August 16, 1992	3	USA (America)	442 (58%)	9–11	Escape, Social isolation, Opposition	Kidcope (Spirito et al., 1988)	Maladjustment	PTSD	Posttraumatic Stress Disorder Reaction Index for Children (RI; Frederick et al., 1992; Lonigan et al., 1991)	M7, M8, M9
Papadatou et al. (2012)	Journal of Traumatic Stress	Wildfire	August, 2007	6	Greece (Europe)	1468 (49%)	12–17	Problem solving, Escape	Kidcope-Adolescent Version (Spirito et al., 1988)	Maladjustment	PTSD, Depression	Children’s Revised Impact of Event Scale (CRIES-13; Smith et al., 2003); Depression Self-Rating Scale (DSRS; Birleson, 1981)	M10, M11, M12
Pina et al. (2008)	Journal of Clinical Child & Adolescent Psychology	Hurricane	August 23, 2005	6	USA (America)	46 (39%)	11	Escape	Children’s Coping Strategies Checklist’s (CCSC; Program for Prevention Research, 1999)	Maladjustment, Adjustment	PTSD, Depression, Anxiety and fear, Social	PTSD Checklist (Amaya-Jackson et al., 1995); Revised Child Anxiety and Depression Scale (RCADS; Chorpita et al., 2000); Family Support Scale (FSS; Dunst et al., 1984)	M13, M14, M15 A22, A23, A25
Qin et al. (2016)	Journal of Traumatic Stress	Earthquake	May 12, 2008	30	China (Asia)	1573 (54%)	15	Social support	Strengths and Difficulties Questionnaire (SDQ; Goodman, 1997)	Maladjustment	PTSD, Depression, Anxiety and fear	PTSD Self-Rating Scale (PTSD-SS; Liu et al., 1998); Depression Self-Rating Scale for Children (DSRSC; Birleson, 1981); Child Anxiety Related Emotional Disorders (SCARED; Birmaher et al., 1997)	M16, M17, M18
Raccanello et al. (2017)	PLoS One	Earthquake	May 20–29, 2012	24	Italy (Europe)	65 (52%)	7–11	Self-reliance	Emotion Regulation Checklist (ERC; Molina et al., 2014)	Adjustment	Emotional	Test of Emotion Comprehension (TEC; Albanese & Molina, 2008)	A36
Russoniello et al. (2002)	Behavioral Medicine	Hurricane	September 7, 1999	6	USA (America)	218 (57%)	9–12	Problem solving, Self-reliance, Social support, Accommodation, Helplessness, Escape, Delegation, Social isolation, Opposition	Kidcope (Spirito et al., 1988)	Maladjustment	PTSD	Post-Traumatic Stress Reaction Index—Child (CPTS-RI; Frederick et al., 1992)	M19, M20, M21, M22, M23, M24, M25, M26, M27, M28
Stratta et al. (2013)	Personality and Individual Differences	Earthquake	April 6, 2009	10	Italy (Europe)	343 (40%)	17–18	Problem solving, Self-reliance	Brief Cope (Carver, 1997)	Adjustment	General	Resilience Scale for Adolescents (READ; Hjemdal et al., 2006)	A5, A6
Terranova et al. (2009)	Journal of Applied Developmental Psychology	Hurricane	August 23, 2005	6	USA (America)	152 (54%)	10–12	Problem solving, Escape	Self-Report Coping Measure (SRCM; Causey & Dubow, 1992); How I Coped Under Pressure Scale (HICUPS; Program for Prevention Research, 1999)	Maladjustment	PTSD, Anxiety and fear	The Child Posttraumatic Stress Disorder Checklist (PTSD Checklist; Amaya-Jackson et al., 1995); Early Adolescent Temperament Questionnaire short form (EATQ; Capaldi & Rothbart, 1992; Ellis & Rothbart, 2001)	M29, M30, M31, M32
Vigil and Geary (2008)	Journal of Family Psychology	Hurricane	October 29, 2005	2	USA (America)	50 (73%)	12–17	Self-reliance, Social support, Accommodation	Family Crisis Oriented Personal Evaluation Scale (F-COPES; McCubbin et al., 1987)	Maladjustment	Depression, Anxiety and fear, Psycho-social distress	Center for Epidemiologic Studies Depression Scale (CES-D; Radloff, 1977); Revised Children’s Manifest Anxiety Scale (RCMAS; Reynolds & Richmond, 1978); Impact of Events Scale-Revised (IES-R; Weiss & Marmar, 1997)	M33, M34, M35, M36, M37, M38, M39, M40, M41
Vigna et al. (2010)	Journal of Black Psychology	Hurricane	August 23, 2005	7	USA (America)	261 (51%)	8–16	Escape	Kidcope (Spirito et al., 1988)	Maladjustment	Depression, Anxiety and fear, Psycho-social distress	Behavioral Assessment System for Children, Second Edition (BASC-2; Reynolds & Kamphaus, 2004)	M42, M43, M44
Wang and Gan (2011)	Anxiety, Stress, & Coping	Earthquake	May 12, 2008	3	China (Asia)	219 (57%)	15–19	Problem solving, Accommodation, Escape	COPE Inventory (Carver et al., 1989)	Maladjustment	Depression	Self-Rating Depression Scale Child (CPTS-RI; Zung et al., 1965)	M45, M46, M47
Wang et al. (2020)	European Journal of Psychotraumatology	Earthquake	April 20, 2013	54	China (Asia)	234 (57%)	11–18	Submission	Event-Related Rumination Inventory (Cann et al., 2011)	Maladjustment, Adjustment	PTSD, General	PTSD checklist for DSM-5 (PCL-5; Weathers, 2013); Posttraumatic Growth Inventory (PTGI; Zhou et al., 2014)	M62 A35
Wu et al. (2014)	Nursing Research	Earthquake	May 12, 2008	3	China (Asia)	1976 (54%)	12–20	Problem solving, Self-reliance, Accommodation, Escape	The Coping Styles Scale (Huang et al., 2000)	Adjustment	Motivational	Self-Concept Scale (TSCS; Fitts, 1965)	A10, A11, A12, A13, A16
Yang et al. (2010)	Social Behaviour and Personality	Earthquake	May 12, 2008	18	China (Asia)	167 (51%)	18	Problem solving, Self-reliance	Coping Style Scale for Middle School Students (CSS-MSS; Chen et al., 2000)	Maladjustment, Adjustment	Psycho-social distress, Social, Motivational,	Symptom Checklist 90 (SCL-90; Derogatis, 1975); Social Support Rating Scale (SSRS; Xiao & Yang, 1987); Generalized Perceived Self-Efficacy Scale (GSES; Schwarzer & Jerusalem, 1995)	M48 A15, A17, A26, A27, A28, A29
Zhang et al. (2014)	PLoS One	Earthquake	May 12, 2008	17	China (Asia)	1420 (57%)	12–20	Problem solving, Self-reliance, Accommodation, Escape	Coping Styles Scale (Huang et al., 2000)	Maladjustment	PTSD	PTSD Checklist-Civilian Chinese Version (PCL-C; Silva, 2017)	M49, M50, M51, M52
Zheng et al. (2012)	PLoS One	Earthquake	May 12, 2008	6	China (Asia)	2250 (54%)	11–18	Self-reliance, Escape	Simplified Coping Style Questionnaire (SCSQ; Xie, 1998)	Maladjustment	PTSD	Posttraumatic Stress Disorder Self-Rating Scale (PTSD-SS; Liu et al., 1998)	M53, M54
Zhou et al. (2015)	Psychological Trauma: Theory, Research, Practice, and Policy	Earthquake	May 12, 2008	54	China (Asia)	354 (53%)	14–20	Submission	Event Related Rumination Inventory (ERRI; Cann et al., 2011)	Maladjustment	PTSD	Child PTSD Symptom Scale (CPSS; Foa et al., 2001)	M55, M56
Zhou and Wu (2016)	Journal of Affective Disorders	Earthquake	April 20, 2013	18	China (Asia)	310 (50%)	12–19	Submission	Event Related Rumination Inventory (ERRI; Cann et al., 2011)	Maladjustment	PTSD	Child PTSD Symptom Scale (CPSS; Foa et al., 2001)	M57, M58
Zhou et al. (2019)	Psychological Trauma: Theory, Research, Practice, and Policy	Earthquake	August 8, 2018	12	China (Asia)	373 (60%)	12–19	Problem solving, Self-reliance	The Coping Style Scale for middle school students (Chen et al., 2000)	Maladjustment, Adjustment	PTSD, General	PTSD Checklist for DSM-5 (Weathers, 2013); Posttraumatic Growth Inventory (PTGI; Zhou et al., 2014)	M59, M60 A33, A34

In line with previous meta-analyses on the efficacy of coping strategies (Clarke, 2006; Cofini et al., 2015; Compas et al., 2001; Fields & Prinz, 1997; Grych & Fincham, 1997), along with studies on the relation between coping strategies and psychological consequences of disasters (Bokszczanin, 2012; Papadatou et al., 2012; Prati & Pietrantoni, 2009; Stratta et al., 2013; Tang et al., 2014; Terranova et al., 2009; Vigil & Geary, 2008; Wang & Gan, 2011), taking into account Zimmer-Gembeck and Skinner’s classification (2011), we hypothesized that after natural disasters: (1) children and adolescents’ use of strategies such as helplessness, escape, delegation, social isolation, submission, and opposition is related to maladjustment (Hypothesis 1); and (2) children and adolescents’ use of problem solving, information seeking, self-reliance, social support (including both seeking and giving), accommodation, and negotiation is related to adjustment (Hypothesis 2). In brief, we examined the efficacy of 12 types of families of coping strategies on measures of maladjustment and adjustment separately by conducting two meta-analyses.

We considered the variety of post-disaster reactions related to maladjustment/adjustment (Celebi Oncu & Metindogan Wise, 2010; Cuzzolaro & Frighi, 1991; Gökçen et al., 2013; Green et al., 1992; Kar, 2009; La Greca et al., 1996; Masten & Osofsky, 2010; Masten et al., 1990; Osofsky, 2004; Prati & Pietrantoni, 2009; Raccanello et al., 2017; Silverman & La Greca, 2002; Vezzali et al., 2016). We explored the moderating role of factors such as age (children, adolescents), type of disasters (earthquakes, floods and hurricanes, wildfires), and continent (America, Europe, Asia).

This work is part of a larger project aimed at understanding the links between emotions and coping strategies in children, adolescents, and adults faced with natural and technological disasters (HEMOT® project, Helmet for EMOTions, https://www.hemot.eu; Raccanello et al., 2019b, 2020a, 2020b, 2020c, 2021; Vicentini et al., 2020). Knowing which coping strategies are effective for diminishing maladjustment and increasing adjustment in children and adolescents is of critical relevance for planning interventions to support victims before, during, and after a disaster.

## Method

### Literature Search and Search Results

**Fig. 1:**
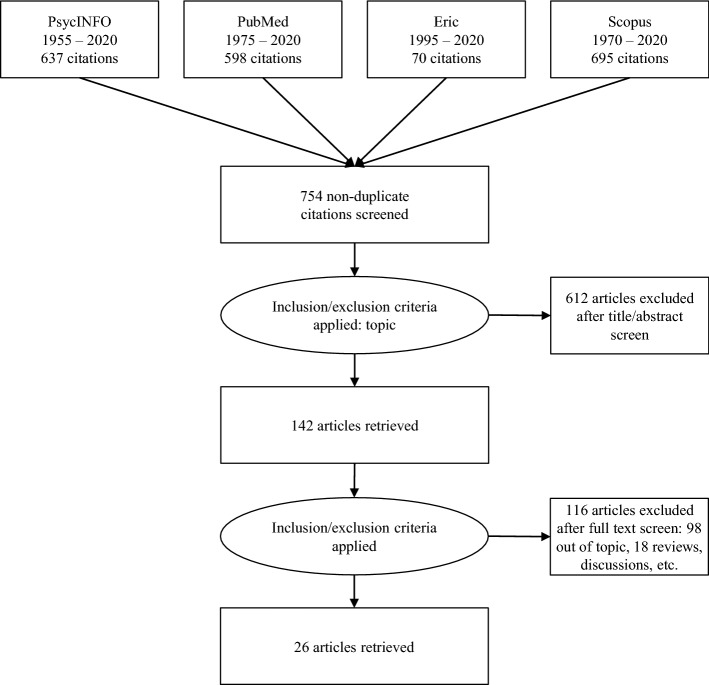
PRISMA Diagram (Moher et al., 2009)

We searched for journal articles explicitly focused on the effects of the exposure to natural disasters amongst children and adolescents. We conducted the literature search during 2020, using the databases PsycINFO, PubMed, Eric, and Scopus. The search terms were: natural disasters, coping, children or adolescents. We report in the PRISMA diagram (Fig. 1) the results of the search strategies and the selection processes (Moher et al., 2009).

Studies included in the meta-analyses: (a) involved participants 18 years of age or younger who had been exposed to a natural disaster; (b) examined at least one coping strategy used to manage the psychological consequences of the disaster; (c) included the assessment of at least one measure of maladjustment and/or one measure of adjustment; (d) reported sufficient statistical information so that effect sizes could be calculated; (e) analyzed data that were collected after the disaster, between two and 54 months after the exposure; (f) were written in English.

We excluded publications reporting reviews, discussions, single-case studies, and qualitative studies. In addition, we excluded those studies which did not include the statistical indexes necessary as inputs for a meta-analysis.

As reported in the PRISMA diagram (Fig. 1), the initial search identified a total of 2000 publications. Six hundred and thirty-seven publications were indexed in PsycINFO and had been published between 1955 and 2020; 598 were indexed in PubMed and had been published between 1975 and 2020; 70 were indexed in Eric and had been published between 1995 and 2020; and 695 were indexed in Scopus and had been published between 1970 and 2020. As a first step, we removed 1246 duplicates from this initial set, i.e., the same publications downloaded in different searches. Then, we screened the 754 selected publications. As a second step, we read all the titles and abstracts and included only the publications pertinent in terms of topic—i.e., respecting the inclusion criteria—for a total of 142. As a third step, we read each article, and that led to us excluding 98 publications because they were off topic, and 18 because they reported reviews, discussions, single-case studies, and qualitative studies. This last step of the selection process was conducted by two independent judges; the reliability was 100%. No publications were excluded after the discussion between judges. Thus, the search identified a selection of 26 publications.

For ethical issues, we adhered to the recommendations of the American Psychological Association.

### Coding and Reliability

We reviewed and coded the eligible studies for several variables. First, we coded the type of family of coping strategy, and we identified whether the studies included maladjustment/adjustment measures. Second, we coded them for other moderating variables, i.e., age group (children: younger than 12 years; adolescents: older than 12 years), type of disaster (earthquakes, floods and hurricanes, wildfires), and continent (America, Europe, Asia). In five studies (Andrades et al., 2018; Cryder et al., 2006; Felton et al., 2013; Pina et al., 2008; Vigna et al., 2010) the data concerning children and adolescents were not separated, so we excluded these studies from the analyses examining the moderating effect of age. Finally, the participants in the studies by Wang et al. (2013) and Zheng et al. (2012) were considered as adolescents even if their age ranged from 11 to 18 years.

For an overview of the included studies, see Table 1.

#### Type of Coping Strategies

We defined coping strategies in line with Lazarus and Folkman (1984), according to whom coping includes drawing upon a set of cognitive and behavioral resources to manage the demands of external circumstances. To distinguish different types of coping strategies relating to children and adolescents, we adapted the classification proposed by Zimmer-Gembeck and Skinner (2011), who assumed a developmental perspective. We coded coping into 12 families: (1) Problem solving, which consists of strategizing, instrument action, and planning; (2) Information seeking, which involves activities like reading, observation, and asking others; (3) Helplessness, which implies confusion, cognitive interference, and cognitive exhaustion; (4) Escape, which regards behavioral avoidance, mental withdrawal, denial, and wishful thinking; (5) Self-reliance, which consists of emotion regulation, behavior regulation, emotional expressions, and emotion approach; (6) Social support, which can be given or received; (7) Delegation, which implies maladaptive help seeking, complaining, whining, and self-pity; (8) Social isolation, which includes social distancing and withdrawal from others; (9) Accommodation, which is a way to distract oneself, minimize, or accept the situation, and can involve a process of cognitive restructuring; (10) Negotiation, which involves attempts of bargaining and persuasion; (11) Submission, which is an attitude of renunciation concerning the problem, and includes rumination in terms of negative repetitive thoughts on the stressful event, rigid perseveration, and intrusive thoughts; (12) Opposition, which implies aggression, lack of cooperation, and blaming others. For each study, we report the instruments used to measure the coded coping strategies in Table 2.

**Table 2:** Functions of Coping Strategies and Description (Adapted From Zimmer-Gembeck & Skinner, 2011)

Adaptive function	Coping strategies	Description
Competence	Problem solving	Concentrating on the problem, aiming at changing the situation to find a solution
	Information seeking	Searching for information
	Helplessness	Giving up, being passive, or confused in front of the requests
	Escape	Avoiding the problem, through behaviors or cognitions
Relatedness	Self-reliance	Counting on oneself, through emotional expression and regulation
	Support seeking	Seeking social, concrete, emotional and/or instrumental support
	Support giving	Giving social, concrete, emotional and/or instrumental support
	Delegation	Assigning the responsibility of the solution to others, complaining or self-pitying
	Social isolation	Disengaging from or refusing social interactions
Autonomy	Accommodation	Adapting smoothly to alternatives and focusing on positive aspects
	Negotiation	Seeking new alternatives, such as finding compromises and allocating priorities
	Submission	Giving up, ruminating or with a rigid attitude
	Opposition	Rejecting collaboration or doing the contrary as regards requests

#### Maladjustment/Adjustment Measures

Many studies explored the psychological consequences of exposure to traumatic events, emphasizing their impact on psychological maladjustment. However, some recent researches suggest that they can be also associated with increased resilience through psychological adjustment (Cheng et al., 2014).

We coded each study for whether it included a measure of maladjustment and/or adjustment (Table 1), taking into account the fact that the exposure to natural disasters can lead to serious negative effects for children and adolescents in both the short and long term (Fergusson & Boden, 2014; Furr et al., 2010; Kar, 2009; Masten & Osofsky, 2010; Neria et al., 2008; Tang et al., 2014; Wang et al., 2013; Weissbecker et al., 2008). We considered as indicators of maladjustment the traumatic consequences for mental health, such as PTSD (e.g., Russoniello et al., 2002; Wang et al., 2020), depression (e.g., Felton et al., 2013; Papadatou et al., 2012), anxiety and fear (e.g., Qin et al., 2016; Terranova et al., 2009), and psycho-social distress (e.g., Vigil & Geary, 2008; Yang et al., 2010).

We coded adjustment considering different indicators pertaining to specific domains (i.e., cognitive, emotional, social, and motivational) or as not related to any specific domain. Indicators of cognitive functioning included, for example, cognitive performance and theory of mind abilities (Cadamuro et al., 2015). The emotional domain referred to indicators of emotional competence, such as the understanding of emotions after the exposure to a natural disaster (e.g., Raccanello et al., 2017). The social domain was operationalized, for example, in terms of understanding of oneself and others, or growth of communication and relationship skills (Bokszczanin, 2012; Yang et al., 2010). Regarding the motivational domain, the indicators pertained to perceived competence, self-efficacy, or self-concept (Kilmer & Gil-Rivas, 2010; Wu et al., 2014; Yang et al., 2010). Concerning the general domain, some indicators of adjustment related, for example, to PTG and resilience (Cryder et al., 2006; Kilmer & Gil-Rivas, 2010; Stratta et al., 2013).

#### Reliability

A first judge coded all the selected articles for coping strategies and maladjustment/adjustment measures. A second judge coded 30% of them for reliability. For coping strategies, the Cohen’s *ĸ* was 0.98, while for maladjustment/adjustment measures it was 1. Disagreements were resolved through discussion between judges.

### Data Analysis

We carried out two meta-analyses to explore the relations between coping strategies and maladjustment on the one hand, and adjustment on the other hand. We conducted the statistical analyses using the Metafor package of R, Version 2.1 (R Core Team, 2020). We computed the effect sizes and corresponding 95% confidence intervals (CI) for each study. We reported the effect sizes as correlations between coping strategies and maladjustment/adjustment. According to Cohen’s criteria (1988), *r* less than |.10| are considered as very weak effects; between |.10| and |.30| as weak effects; between |.30| and |.50| as moderate effects; and higher than |.50| as large effects.

An important assumption in traditional meta-analytic approaches is that there is no dependency between effect sizes in the data set. In our data set, in many cases, we had more than one effect size extracted from the same study, thus resulting in interdependent effect sizes. In the literature, there are various suggested approaches for dealing with this interdependency (e.g., Assink & Wibbelink, 2016; Tanner-Smith & Tipton, 2014; Van den Noortgate & Onghena, 2003). We chose the multilevel approach using the rma.mv function of the Metafor package. This solution allowed us to account for the dependency within the studies, assigning the same random effect to effect sizes with the same value of the grouping variable (that is the variable “study” in our work). However, because Van den Noortgate and Onghena (2003) suggested that, for models without moderators, the results of the multilevel approach are not substantially different from the results of the traditional random-effects approaches, we chose to run traditional random-effects meta-analyses to evaluate the main effects, the publication bias, and the presence of outliers, while we used multilevel mixed-effects meta-analyses to evaluate the role of the moderators. We performed the multilevel mixed-effects models using the restricted maximum-likelihood estimation method, in order to take into account non-independent sampling errors due to the presence of multiple effects in the studies (Borenstein, 2009). We examined the impact of each moderator on the effect size using separate mixed-effect models and, at the same time, we accounted for the dependence of effect sizes belonging to the same studies by using multilevel modelling (*level 1* = effect sizes, the variable which identified all effect sizes; *level 2* = study, the variable which identified primary studies). We also calculated intra-class correlation (ICC) to confirm that the multilevel approach was appropriate for our datasets. In multilevel analyses, ICC values higher than 0.05 support the use of a multilevel strategy (LeBreton & Senter, 2008).

We explored the role of the following moderator variables, separately for maladjustment and adjustment:

- Types of coping strategies (problem solving, information seeking, helplessness, escape, self-reliance, social support, delegation, social isolation, accommodation, negotiation, submission, opposition);
- Age (children, adolescents);
- Type of disaster (earthquakes, floods and hurricanes, wildfires);
- Continent (America, Europe, Asia).

Only studies that had information regarding each moderator were included in the corresponding analysis. Furthermore, we analyzed the interaction with the type of coping strategies for age, type of disaster, and continent.

We evaluated heterogeneity across studies by using Cochran’s heterogeneity statistic (*Q*), in order to test the null hypothesis according to which the effect sizes of different studies are similar enough to share a common effect size (Cochran, 1954). There is heterogeneity between the effects if a significant value of *Q* is found. We also used the *Q* statistic to test the significance of moderators (a significant *Q* for the comparison indicates that the difference between the combined effect sizes of the subsets of studies is significant; Borenstein, 2009; Rosenthal, 1995). To verify the level of heterogeneity, we used the *I*^*2*^ statistic, which measures the proportion of total variance due to the variability between studies. Low values of the statistic (i.e., 1–49) correspond to low heterogeneity, medium values (i.e., 50–74) correspond to moderate heterogeneity, and high values (i.e., 75–100) correspond to high levels of heterogeneity. We checked for potential outliers by examining the distribution of the effect sizes (funnel plot and radial plot) and the influence of individual studies on heterogeneity (*Q* statistic) and on the general model (Cook’s distance). To investigate potential publication biases (i.e., biases due to the publication process whereby those studies without significant results are not published), we used the trim and fill approach of Duval and Tweedie (2000). This is a non-parametric method that estimates the number of studies missing from the meta-analysis by suppressing the studies that generate patterns of asymmetry, and generating new data based on the initial sample to obtain a symmetrical effect size distribution. For this analysis, a funnel plot is constructed by plotting the effect size against the standard error for each study.

## Results

We conducted two meta-analyses, studying the relationship between coping strategies and maladjustment in one analysis, and between coping strategies and adjustment in the other.

### Coping Strategies and Maladjustment

**Fig. 2:**
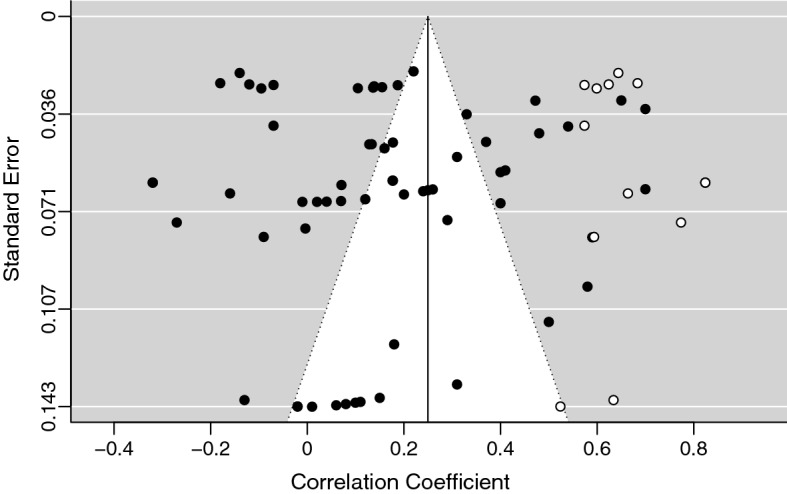
Funnel Plot of Maladjustment Indicators Effect Size. For the Trim-and-Fill Analysis, we Added 13 Studies on the Right Side

**Fig. 3:**
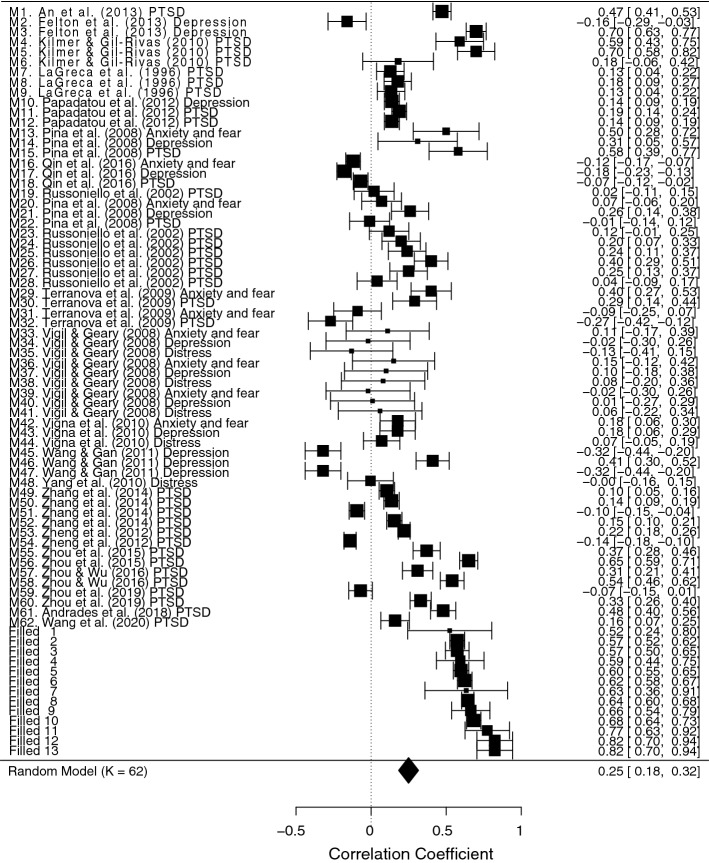
Forest Plot of Maladjustment Effect Size. For Each Study There Were One or More Indicators of Maladjustment (M, see Table 1)

Initially, we analyzed the correlations between coping strategies and maladjustment for the studies included in the meta-analysis. The effect sizes were in different directions for diverse coping strategies, but they seemed to be in the expected direction. The random effects model, *k* = 64, *n* = 9692, estimated a weak medium effect size, *r* = 0.19, 95% CI [0.12, 0.26], *SE* = 0.04. The studies were heterogeneous, *Q*(63) = 22,625.62, *p* < 0.001, and the proportion of total variance due to the variability between the studies was very high, *I*^*2*^ = 98.94%. The trim and fill test was significant, suggesting the presence of a publication bias and indicating the need to add 14 effects on the right side. The new estimated effect size was higher, *r* = 0.28, 95% CI [0.21, 0.36], *SE* = 0.04. Then, we evaluated the presence of potential outliers checking the distribution of the effect sizes (using the funnel plot and the radial plot) and the influence of individual studies on heterogeneity (Q statistic) and on the general model (Cook’s distance). Two studies (i.e., Papadatou et al., 2012; Yang et al., 2010) had an effect size very far from the medium effect size estimated and from the confidence intervals, with *r* = 0.96 and *r* = 0.93, respectively. The radial plot confirmed the anomaly of these effects. Furthermore, both studies had high Cook’s distance values, indicating a huge influence on the medium effect size estimated and had also a large influence on the heterogeneity measure. Consequently, we decided to exclude them and to run a third random-effects model. Again, the trim and fill test was significant, suggesting the presence of a publication bias. The test indicated the need to add 13 effects, on the right side (see Fig. 2). The effect size estimated by this model was still moderate, *r* = 0.25, 95% CI [0.18, 0.32], *SE* = 0.03, although lower but more accurate than that estimated by the first random-effects model (Fig. 3). The heterogeneity of the studies was still very high, *Q*(74) = 3993.05, *p* < 0.001, *I*^2^ = 96.82%. At the end of this process, we checked whether the estimated effect was really close to that obtained by the multilevel random-effects model. The effect size estimated through the multilevel model was effectively close to the effect size of the third model, *r* = 0.23, 95% CI [0.13, 0.33], *SE* = 0.05 (Van den Noortgate & Onghena, 2003). The ICC was 0.34, confirming the importance of using a multilevel analysis.

Then, we ran multilevel mixed-effects models to verify whether some moderator variables could explain the high heterogeneity. Only the type of coping strategies and the continent seemed to moderate the relation between maladjustment and coping strategies. However, the interaction between the type of coping strategies and the other moderators (age of participants, type of disaster, and continent) was statistically significant. The moderator analysis for the type of coping strategies, *Q*_*MODEL*_(10) = 333.94, *p* < 0.001, explained the variance in the primary effect sizes and indicated that the remaining variability across effect sizes was still heterogeneous, *Q*_*RESIDUAL*_(51) = 654.43, *p* < 0.001. The analysis for the type of coping strategies also highlighted a positive statistically significant relation between maladjustment and some coping strategies, i.e., escape, *r* = 0.19, *p* < 0.001, social isolation, *r* = 0.15, *p* = 0.017, submission, *r* = 0.64, *p* < 0.001, and opposition, *r* = 0.16, *p* = 0.009. The moderator analysis for continent, *Q*_*MODEL*_(3) = 21.39, *p* < 0.001, explained the variance in the primary effect sizes and indicated that the remaining variability across effect sizes was still heterogeneous, *Q*_*RESIDUAL*_(51) = 1818.69, *p* < 0.001. Concerning age, we found a statistically significant interaction with coping strategies, *Q*_*MODEL*(16)_ = 247.04, *p* < 0.001. For children there was a positive relation between self-reliance and maladjustment, *r* = 0.34, *p* < 0.001. We also found an interaction between type of coping strategies and type of disaster. In particular, there was a negative relation between maladjustment and escape for wildfires, *r* = − 0.20, *p* = 0.001; for earthquakes, the analysis highlighted a negative relation of maladjustment with problem solving, *r* = − 0.19, *p* = 0.009, self-reliance, *r* = − 0.29, *p* < 0.001, and submission, *r* = − 0.43, *p* < 0.001. Finally, the interaction between coping strategies and continent revealed that in North America problem solving, *r* = 0.20, *p* = 0.007, self-reliance, *r* = 0.29, *p* < 0.001, and submission, *r* = 0.43, *p* < 0.001, were positively associated with maladjustment. In Europe escape resulted negatively associated with maladjustment, *r* = − 0.42, *p* < 0.001.

### Coping Strategies and Adjustment

**Fig. 4:**
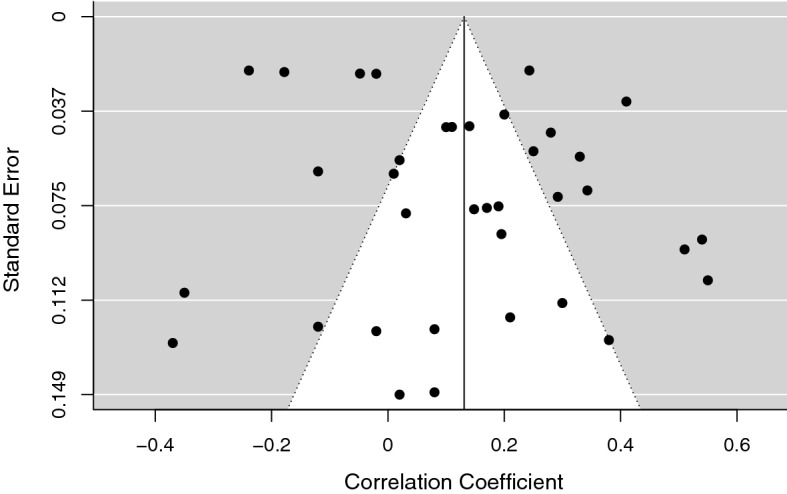
Funnel Plot of Adjustment Effect Size. The Trim-and-Fill Analysis Suggested the Absence of Publication Biases

**Fig. 5:**
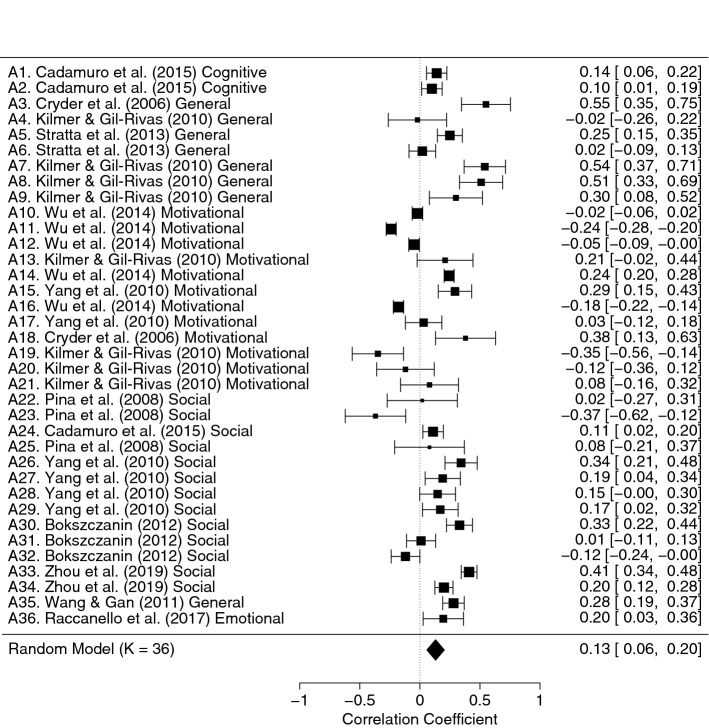
Forest Plot of Adjustment Indicators Effect Size. For Each Study There Were One or More Indicators of Adjustment (A, see Table 1)

Concerning adjustment, the correlations with coping strategies for the individual studies included in the meta-analysis were in different directions for diverse coping strategies, but they seemed to be in the expected direction as well. The random-effects model, *k* = 37, *n* = 3504, estimated a small medium effect size, *r* = 0.15, 95% CI [0.07, 0.22], *SE* = 0.04. The studies were heterogeneous, *Q*(36) = 1194.41, *p* < 0.001, and the proportion of total variance due to the variability between the studies was very high, *I*^2^ = 96.18%. The trim and fill test was not significant, suggesting the absence of publication biases (Fig. 4). Then, again we evaluated the presence of potential outliers checking the distribution of the effect sizes (through the funnel plot and the radial plot) and the influence of individual studies on heterogeneity (Q statistic) and on the general model (Cook’s distance). The analysis of the outliers suggested that the study by Andrades et al. (2018) had to be deleted. In this case, the effect size was very far from the medium effect size estimated and from the confidence intervals, *r* = 0.68. It was characterized by a high Cook’s distance value, and it had a large influence on the studies’ heterogeneity. After the exclusion of this study we reran the model to calculate the new estimated effect size, i.e., *r* = 0.13, 95% CI [0.06, 0.20], *SE* = 0.04. The heterogeneity of the studies was still very high, *Q*(35) = 779.01, *p* < 0.001, *I*^2^ = 95.27% (see the forest plot in Fig. 5). Then, we checked the presence of a publication bias through the trim and fill test, but it was again not statistically significant. We also checked whether the estimated effect was similar to that obtained by the multilevel analysis which took into account the dependence of effect sizes from the same studies. The effect size estimated with the multilevel approach was very close to that of our traditional random-effects model, *r* = 0.17, 95% CI [0.08, 0.26], *SE* = 0.05 (Van den Noortgate & Onghena, 2003). The ICC was 0.16, confirming also for this dataset the relevance of using a multilevel analysis.

We proceeded with the moderation analysis to assess whether moderator variables could explain the high heterogeneity. Only type of coping strategies moderated the relation between coping strategies and adjustment. We also evaluated the interaction between type of coping strategies and the other potential moderators, and we found an interaction for all of them (i.e., type of disaster), except continent. The moderator analysis for type of coping strategies, *Q*_*MODEL*_(5) = 271.13, *p* < 0.001, explained the variance in the primary effect sizes but indicated that the remaining variability across effect sizes was still heterogeneous, *Q*_*RESIDUAL*_(30) = 315.18, *p* < 0.001. The analysis for the type of coping strategies highlighted a positive statistically significant relation between adjustment and some coping strategies, i.e., problem solving, *r* = 0.31, *p* < 0.001, social support, *r* = 0.22, *p* = 0.005, and submission, *r* = 0.30, *p* < 0.001. In the evaluation of the interaction between type of coping strategies and age, even if we found a statistically significant interaction, *Q*_*MODEL*_(10) = 272.24, *p* < 0.001, no correlation was statistically significant separately for children and adolescents. The same happened for the interaction between coping strategies and type of disaster, *Q*_*MODEL*_(8) = 273.28, *p* < 0.001.

## Discussion

These meta-analyses aimed at exploring the efficacy of different disaster-related coping strategies in children and adolescents. Acknowledging that the efficacy of coping depends on a variety of factors pertaining both to individuals and the context in which they find themselves, the results enabled us to identify which coping strategies seem more adequate for helping children and adolescents to face a natural disaster.

The analysis of the studies on maladjustment confirmed a statistically significant and positive relation with some coping strategies, i.e., escape, social isolation, submission, and opposition, supporting Hypothesis 1. According to Zimmer-Gembeck and Skinner (2011), “maladaptive” families of coping strategies have effects on the maintenance and reinforcement of psychopathological symptoms, in particular PTSD, depression, anxiety and fear, connected to disaster exposure. These symptoms have an important role in inhibiting the activation of adaptive strategies, and predispose subjects to dysfunctional ways of coping with stressors, such as avoidance, escape, aggression, social withdrawal, etc. Helplessness was one of the two groups of coping strategies that did not show a significant relation with maladjustment. However, this strategy inhibits any action, while the other strategies involve the activation of maladaptive behaviors. The other group was delegation, which is conceptualized by Zimmer-Gembeck and Skinner (2011) as maladaptive for its focus on self-pity, complaining, or whining. However, it might be that delegating results in a feeling of being relieved of one’s responsibilities and may then possibly play an adaptive role. Future studies should investigate this possibility.

The results show a statistically significant and positive relationship between adjustment and some coping strategies: problem solving, social support, and submission. According to previous studies (Bokszczanin, 2012; Swiatek, 2001; Zimmer-Gembeck & Skinner, 2011), problem solving and social support have an adaptive role as coping families oriented to the activation of effective environmental resources and management of social resources involving relationships. Unexpectedly, submission was positively related to adjustment. According to Skinner et al. (2003), this strategy is considered a “maladaptive” family. It is worth noting that the studies considered in the current meta-analysis explored only the relation between submission and PTG. PTG is a complex construct, in which different factors tend to coexist, such as a greater awareness of personal strength, a change of perspective regarding one’s relationships, a change in the philosophy of life in terms of greater appreciation for life and new possibilities, and spiritual growth (Tedeschi et al., 1998). For example, a recent study investigated the correlation between submission and PTG, emphasizing that discomfort in combination with reflexive processes could facilitate positive changes in the subject’s functioning after exposure to trauma (Kilmer & Gil‐Rivas, 2010). Therefore, Hypothesis 2 was only partially supported.

In addition, we examined the moderating role of age, type of disaster, and continent. The results of the two meta-analyses suggested that some moderators have an effect on the relationships with coping strategies. For example, children show a more positive relation between maladjustment and self-reliance compared with adolescents. Indeed, children have fewer resources available to cope with traumatic events than adolescents because the emotional competence is necessarily related to the child’s developmental growth (Compas, 1987). Furthermore, the results of the analyses revealed the moderating effect of type of disaster. We found a significant and negative relation between maladjustment and escape in the case of wildfires. In this case, using a behavioral escape strategy can be adaptive. If this strategy is activated immediately, the negative symptoms associated with the disaster could be inhibited and mitigated by the shorter duration of exposure. In line with the literature (Zimmer-Gembeck & Skinner, 2011), children and adolescents exposed to an earthquake showed a negative relation between maladjustment and problem solving. Finally, we explored the interaction between coping strategies and continent. In North America, submission and, unexpectedly, problem solving and self-reliance strategies were positively associated with maladjustment; in Europe, escape resulted negatively associated with maladjustment. Although this result is ambiguous, it is necessary to underline the high heterogeneity of the studies, with an unequal distribution of the disasters across the three continents investigated. Future research should examine this issue. Finally, no significant moderators were found for adjustment; we could speculate that this was due to the small number of studies available for analysis.

Our study suffers from several limitations. First, the studies available were characterized by a high heterogeneity. For example, some studies focused on only maladjustment or adjustment measures, while others focused on both. It was also the case that studies included in these meta-analyses varied considerably in the definition of coping strategies, in the terminology used, and in the instruments employed to assess them. Future research could focus on these issues including, for example, studies concerning technological disasters. Second, the number of studies selected through the PRISMA was relatively small. Consequently, we could not directly investigate the differential impact of each category of maladjustment (four categories) and adjustment (five categories) on each coping strategy (12 types) because the number of studies for each intersection was quite low and the data was non-homogeneous. Third, we did not examine the effects of moderators such as gender or time from the disaster.

These meta-analyses does help to clarify which coping strategies are the most effective in diminishing and/or avoiding traumatic consequences of natural disasters in children and adolescent victims. This knowledge can be the base from which to develop actions focused on increasing awareness about and implementation of effective strategies amongst both professionals and the public. A central issue is creating content that is relevant for children and adolescents; that deals with emotions and their regulation and provides tools that can be used, taking account of the characteristics of each type of disaster. A related issue is developing ways in which the content might be disseminated. Computer-based systems have already been developed to allow for rapid geographically dispersed delivery of content (Raccanello & Burro, 2019).

Studies have shown that training children in coping strategies can have benefits in the event of an earthquake (Raccanello et al., 2020b). The efficacy of this kind of training, using evidence-based techniques, in advance of a disaster can be demonstrated (Flay et al., 2005). During an emergency, when timing is critical, public communication campaigns can be used to deliver information to people faced with helping children confront the looming disaster. An example was the use of the Internet to distribute a pamphlet designed to help young people cope with the emotional impact of the COVID-19 pandemic (Raccanello et al., 2020c). After a disaster, these same techniques can be paired with other psychological support methods to aid in recovery.
